# Label-free quantitative phosphorylation analysis of human transgelin2 in Jurkat T cells reveals distinct phosphorylation patterns under PKA and PKC activation conditions

**DOI:** 10.1186/s12953-015-0070-9

**Published:** 2015-03-26

**Authors:** Se Hwan Jang, Chang-Duk Jun, Zee-Yong Park

**Affiliations:** School of Life Sciences, Gwangju Institute of Science & Technology, 123, Cheomdangwagi-Ro, Buk-Gu, 500-712 Gwangju Republic of Korea

**Keywords:** Label-free, Relative quantification, PKA, PKC, Phosphorylation, LC-MS/MS, Immune response activation, Immune response homeostasis

## Abstract

**Background:**

Transgelin2, one of cytoskeletal actin binding proteins has recently been suggested to be involved in the formation of immune synapses. Although detailed function of transgelin2 is largely unknown, interactions between transgelin2 and actin appear to be important in regulating cellular functions of transgelin2. Because protein phosphorylation can change ability to interact with other proteins, comprehensive phosphorylation analysis of transgelin2 will be helpful in understanding its functional mechanisms.

**Results:**

Here, a specific protein label-free quantitative phosphorylation analysis method combining immuno-precipitation, IMAC phosphopeptide enrichment technique and label-free relative quantification analysis was used to monitor the phosphorylation changes of transgelin2 overexpressed in Jurkat T cells under protein kinase C (PKC) and protein kinase A (PKA) activation conditions, two representative intracellular signalling pathways of immune cell activation and homeostasis. A total of six serine/threonine phosphorylation sites were identified including threonine-84, a novel phosphorylation site. Notably, distinct phosphorylation patterns of transgelin2 under the two kinase activation conditions were observed. Most phosphorylation sites showing specific kinase-dependent phosphorylation changes were discretely located in two previously characterized actin-binding regions: actin-binding site (ABS) and calponin repeat domain (CNR). PKC activation increased phosphorylation of threonine-180 and serine-185 in the CNR, and PKA activation increased phosphorylation of serine-163 in the ABS.

**Conclusions:**

Multiple actin-binding regions of transgelin2 participate to accomplish its full actin-binding capability, and the actin-binding affinity of each actin-binding region appears to be modulated by specific kinase-dependent phosphorylation changes. Accordingly, different actin-binding properties or cellular functions of transgelin2 may result from distinct intracellular signalling events under immune response activation or homeostasis conditions.

**Electronic supplementary material:**

The online version of this article (doi:10.1186/s12953-015-0070-9) contains supplementary material, which is available to authorized users.

## Background

Transgelin2 was characterized as a smooth muscle cytoskeletal protein and subsequently classified into the calponin family. Although the detailed function of transgelin2 is largely unknown, co-localization with F-actin cytoskeleton in the cytoplasm has been recently reported [1]. Close homologues of transgelin2 such as transgelin/SM22α and calponin1 have been studied much more extensively than transgelin2 and possess actin-binding properties [2-6]. Functional studies of transgelin/SM22α and calponin1 have shown that these transgelin2 homologues are involved in actin cross-linking/ gelling process. These observations suggest that transgelin2 may also participate in the regulation of actin cytoskeleton dynamics. Because several proteomics studies have suggested transgelin2 as a potential biomarker candidate of tumorigenesis and metastasis, the functional roles of transgelin2 have predominantly been investigated using various cancer cell lines and tissue samples [7-9]. Interestingly, research groups have reported somewhat contradictory results regarding the expression levels and cellular functions of transgelin2. Both oncogenic and tumor suppressive effects of transgelin2 have been observed depending on the types of cancer cell lines and tissue samples investigated. For example, Zhang et al. and Rho et al. reported the increased expression of transgelin2 in colorectal cancer and lung adenocarcinoma patient tissue samples, respectively [7,9]. Elsner et al. observed down-regulated transgelin2 expression in Barrett’s adenocarcinoma patient tissue samples [10]. Regarding cancer cell lines, Yoshino et al. reported an oncogenic function of transgelin2 in bladder cancer cell lines [11]. These oncogenic functions of transgelin2 were further confirmed in other types of cancer cells, including lung squamous carcinoma cells, renal carcinoma cells, and head and neck squamous carcinoma cells by the same research group [12-15]. In contrast, tumor-suppressive function and anti-angiogenesis effects of transgelin2 were observed in hepatocyte cells and endothelial cells, respectively [1,16]. Because there is no simple explanation for these contradictory results, additional properties in addition to the changes in the expression level of transgelin2 must exist. Recently, Leung et al. reported transgelin2 as one of the downstream signal transduction molecules of PFTK1 protein kinase in the control of liver cancer cell motility [16]. They suggested that the phosphorylation of serine-83 and serine-163 of transgelin2 plays an important role in controlling cancer cell invasion and motility. Although the connection between specific amino acid phosphorylation and the tumor suppressor function of transgelin2 was established by site-directed mutagenesis (phosphorylation-mimetic and -defective) in their study, evidence for the actual phosphorylation of these two amino acid residues was not provided. A more focused and comprehensive protein quantitative phosphorylation analysis that can monitor phosphorylation changes of individual transgelin2 phosphorylation sites under different conditions is necessary to consolidate the functional relevance of transgelin2 phosphorylation. A quantitative phosphorylation analysis focusing on transgelin2 has not been previously conducted; however, several large-scale quantitative phosphoproteomics studies have reported phosphorylation level changes of transgelin2 in immune cells [17-19]. Because these studies investigated system-wide signal transduction pathways in different types of T cells, only one or two phosphorylation sites of transgelin2 were quantitatively analysed. To reveal the functional significance of transgelin2 phosphorylation events, comprehensive transgelin2 quantitative phosphorylation analysis under more specific kinase activation conditions is required.

In this study, a specific protein label free quantitative phosphorylation analysis method combining immuno-precipitation, IMAC phosphopeptide enrichment technique and mass spectrometric label-free relative quantitation was used to achieve comprehensive phosphorylation analysis of transgelin2 under any given conditions. While only two phosphorylation sites of transgelin2 were investigated in large-scale quantitative phosphorylation analyses of immune cells, five phosphorylation sites of transgelin2 were quantitatively analysed in this study. Reproducibility of the entire sample preparation procedure was first systematically evaluated. Our method was then used to investigate the phosphorylation changes of transgelin2 overexpressed in Jurkat T cells under two different kinase activation conditions. Jurkat T cells are a well-characterized model for studying immune response signaling. Protein kinase A (PKA) and protein kinase C (PKC) activation conditions were chosen in this study as the two representative serine/threonine kinase cascades of immune response homeostasis and activation, respectively [20,21]. We hypothesized that the serine/threonine phosphorylation events of transgelin2 are discretely modulated by specific kinases that were activated under immune response activation or homeostasis conditions, resulting in two distinctive transgelin2 functions. To our knowledge, the functional relevance of transgelin2 phosphorylation in immune cell responses has not been described previously.

## Results

### The phosphatase inhibitory effects of calyculin A

**Figure 1:**
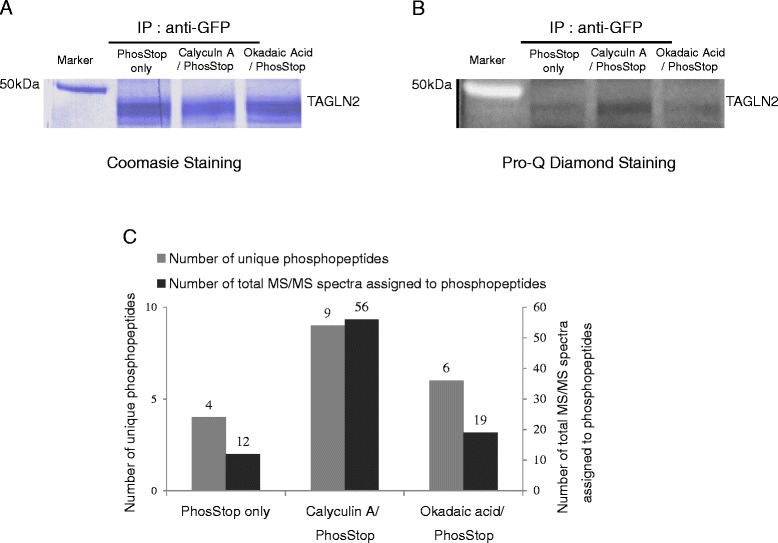
**Calyculin A treatment effects in the phosphorylation analysis of transgelin2.** ProQ-Diamond and Coomassie sequential staining results of GFP tagged transgelin2 following SDS-PAGE separation **(A and B)**, and micro RPLC-MS/MS analysis results **(C)**.

Conventional phosphatase inhibitor treatments of the cell lysates such as phosphatase inhibitor cocktails did not effectively improve the identification of transgelin2 Ser/Thr phosphorylation sites. Only one or two phosphorylation sites were typically identified. To increase the number of transgelin2 phosphorylation sites identified in the quantitative phosphorylation analysis, various phosphatase inhibitors such as calyculin A and okadaic acid were tested. Among the phosphatase inhibitors tested in our laboratory, calyculin A treatment exhibited the most significant changes in terms of the total numbers of identified phosphorylation sites and phosphopeptides. To show the phosphatase inhibitory effects of calyculin A, Jurkat T cells overexpressing GFP-tagged transgelin2 were treated with calyculin A for 30 min, okadaic acid for 30 min or untreated prior to cell harvest. These three cell samples were lysed separately with lysis buffers containing conventional phosphatase inhibitors, followed by immuno-precipitations with anti-GFP antibodies. The immuno-purified GFP-tagged transgelin2 samples were then subjected to SDS-PAGE and the resulting SDS-PAGE gel was sequentially stained with Pro-Q Diamond and Coomassie. The Coomassie staining results showed one major protein band that matched the molecular weight of GFP-tagged transgelin2, and the intensities of this protein band were not noticeably different among the untreated and two phosphatase-treated conditions (Figure 1A). In contrast, the Pro-Q Diamond staining results indicated significantly higher phosphorylation levels of transgelin2 in calyculin A-treated samples (Figure 1B). The increased phosphorylation level of the GFP-tagged transgelin2 in calyculin A-treated sample was further confirmed by micro RPLC-MS/MS analysis. The number of unique phosphopeptide identified was increased from 4 to 9 and a total of 6 phosphorylation sites were identified in the samples with the calyculin A treatment. The total number of MS/MS spectra assigned to phosphopeptides was increased by more than four-fold from 12 to 56 through the use of the calyculin A treatment (Figure 1C). Among the phosphorylation sites identified in the calyculin A-treated sample, threonine-84 has not been previously identified by mass spectrometry. MS/MS spectrum of the phosphopeptide containing threonine-84 was manually validated, and the results are shown in Additional file 1: Figure S1.

### Reproducibility of a specific protein label-free quantitative phosphorylation analysis

To demonstrate our reproducible phosphopeptide sample preparation procedure, three aliquots of cell lysates containing 5 mg proteins were independently subjected to immuno-precipitation, SDS-PAGE, in-gel digestion, and the IMAC phosphopeptide-enrichment processes. Table 1 shows the micro RPLC-MS/MS analysis results from three independent experiments. Several phosphopeptides that were commonly identified in all three independent experiments are not included in Table 1 because the signal-to-noise ratios of the selected ion chromatograms were too low for peak area comparison. Although the peak areas of these phosphopeptides differed by more than two orders of magnitude, the relative standard deviations of each phosphopeptide ranged from 1.6 to 20.4%, with an average relative standard deviation of 9.5%. Because most label-free quantitative phosphorylation analysis studies employ peak area normalization using co-injected phosphopeptide standards or non-phosphopeptides in the sample, the effects of phosphopeptide peak area normalization were tested using co-added bovine alpha casein tryptic digest peptides. The phosphopeptide peak area normalization results are shown in Additional file 2: Table S1. There were no significant differences observed before and after phosphopeptide peak area normalizations, as the average relative standard deviations was 10.0%. The linearity of our quantitative phosphorylation analysis was also tested using a standard protein, bovine alpha casein. Various concentrations of alpha casein samples ranging from 0.1 to 1.0 μg were added to the SDS-PAGE separation (Additional file 3: Figure S2A). Three independent experiments were performed and the peak areas of the individual alpha casein phosphopeptides were plotted against the loaded amounts of alpha casein (Additional file 3: Figure S2B, C, D). A linear response of peak area measurements was observed in the concentration range tested for all three phosphopeptides and the R^2^ values ranged from 0.9851 to 0.9982.

**Table 1:** **Reproducibility of specific protein label-free quantitative phosphorylation analyses from three independent experiments**

**Human transgelin2 tryptic digest phosphopeptides identified**	**Site**	**Calyculin A treated Sample 1**	**Calyculin A treated Sample 2**	**Calyculin A treated Sample 3**	**Avg(Relative. Std)**
GPAYGL**pS**R(+2)	11th	62.48 × E06	60.18 × E06	71.39 × E06	**64.68 × E06 (0.092)**
GPAYGL**pS**REVQQK(+2)	11th	27.16 × E06	27.77 × E06	26.94 × E06	**27.29 × E06 (0.016)**
IQAS**pTM(ox)**AFK(+2)	84th	2.41 × E06	2.72 × E06	2.80 × E06	**2.64 × E06 (0.078)**
NF**pS**DNQLQEGK(+2)	163th	168.18 × E06	193.05 × E06	186.64 × E06	**182.62 × E06 (0.071)**
NF**pS**DNQLQEGK(+1)	163th	3.46 × E06	4.93 × E06	4.13 × E06	**4.17 × E06 (0.176)**
NVIGLQMG**pT**NR(+2)	180th	1.94 × E06	1.46 × E06	1.96 × E06	**1.79 × E06 (0.158)**
NVIGLQ**M(ox)**G**pT**NR(+2)	180th	1.97 × E06	2.52 × E06	2.38 × E06	**2.29 × E06 (0.125)**
GA**pS**QAGMTGYGMPR(+2)	185th	62.43 × E06	55.27 × E06	61.93 × E06	**59.88 × E06 (0.067)**
GA**pS**QAG**M(ox)**TGYGMPR(+2)	185th	73.58 × E06	80.29 × E06	77.44 × E06	**77.10 × E06 (0.044)**
GA**pS**QAGMTGYG**M(ox)**PR(+2)	185th	43.92 × E06	43.28 × E06	42.38 × E06	**43.19 × E06 (0.018)**
GA**pS**QAG**M(ox)**TGYG**M(ox)**PR(+2)	185th	72.91 × E06	82.53 × E06	107.47 × E06	**87.64 × E06 (0.204)**

Three independent label-free quantitative phosphorylation analyses of transgelin2 were carried out to show the reproducibility of entire sample preparation procedure. Selected ion chromatograms of phosphopeptides were constructed from three micro RPLC-MS/MS analyses, and peak areas were plotted.

### Quantitative phosphorylation analysis of transgelin2 under PKC and PKA activation conditions

**Figure 2:**
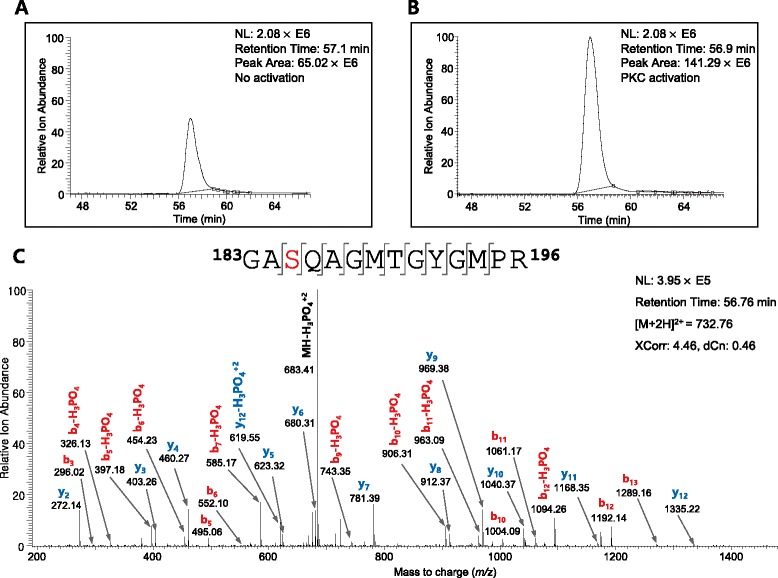
**PKC-dependent phosphorylation changes of transgelin2 serine-185.** Selected ion chromatograms of a phosphopeptide containing serine-185 under no activation **(A)** and PKC activation **(B)** conditions. Manually assigned MS/MS spectrum of phosphopeptide containing serine-185 **(C)**.

**Figure 3:**
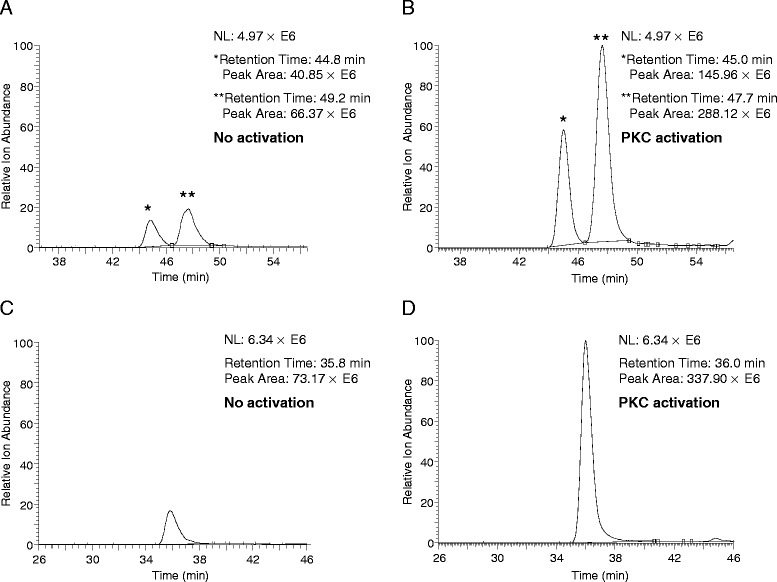
**PKC-dependent phosphorylation changes of transgelin2 serine-185.** Selected ion chromatograms of two isobaric phosphopeptides containing one oxidized methionine (^183^GApSQAGM^ox^TGYGMPR^196^ and ^183^GApSQAGMTGYGM^ox^PR^196^) **(A and B)** and the same sequence phosphopeptide with two oxidized methionines under no activation and PKC activation condition **(C and D)**. * denotes ^183^GApSQAGMTGYGM^ox^PR^196^ and ** denotes ^183^GApSQAGM^ox^TGYGMPR^196^.

**Figure 4:**
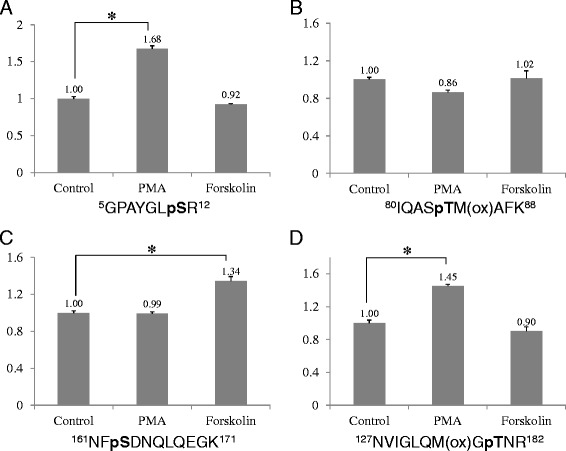
**Label-free relative quantitative phosphorylation analysis results of transgelin2 phosphorylation sites under PKA and PKC activation conditions.** The peak areas of the individual phosphopeptides containing ser-11 **(A)**, threonine-84 **(B)**, serine-163 **(C)** and threonine-180 **(D)** from the three independent experiments under the above two kinase activation conditions were calculated and plotted in terms of the peak area ratios compared with the no activation condition experiments. * denotes the level of statistically significant difference in three independent sets of experiments. (p < 0.05).

Our label-free quantitative phosphorylation analysis method was then used to investigate the phosphorylation level changes of GFP-tagged transgelin2 overexpressed in Jurkat T cells under PKC activation conditions. The most significant phosphorylation level change was observed with serine-185, in which the phosphorylation increased by more than two-fold. A manually validated MS/MS spectrum and selected ion chromatograms of the phosphopeptide containing serine-185 with or without PMA treatment are shown in Figure 2. Other phosphorylation sites showing significant phosphorylation level changes included serine-11 and threonine-180 (Additional file 4: Figure S3 and Additional file 5: Figure S4). Additionally, phosphopeptides containing methionine residue(s) were identified as discrete phosphopeptides, depending on the numbers and locations of oxidized methionine. For example, serine-185 co-localized with two methionine residues in the same tryptic digest peptide was identified in four different phosphopeptides. Two isobaric phosphopeptides, ^183^GAS*QAGM^ox^TGYGMPR^196^ and ^183^GAS*QAGMTGYGM^ox^PR^196^, whose only difference consisted of the location of the oxidized methionine, were clearly separated in the RPLC separation and unambiguously identified (Figure 3A and B). Finally, selected ion chromatograms of the phosphopeptides containing serine-185 with two oxidized methionines are shown in Figure 3C and D. The phosphorylation of transgelin2 under the PKA activation condition was also investigated by our quantitative phosphorylation analysis method. Although the number of phosphorylation sites and phosphopeptides identified were identical compared with those from the PKC activation, a moderate level of phosphorylation change (~36% increase) was observed only at serine-163 (Additional file 6: Figure S5). To increase the confidence level of our transgelin2 quantitative phosphorylation analysis results, the PKA, PKC and no activation condition experiments were independently performed three times. The peak areas of the individual phosphopeptides from the three independent experiments under the above two kinase activation conditions were calculated and plotted in terms of the peak area ratios compared with the no activation condition experiments (Figure 4 and Additional file 7: Figure S6). The preferential phosphorylation sites of transgelin2 under the PKA and PKC activation conditions appeared to vary. Serine-11, threonine-180 and serine-185 were responsive solely to PKC activation, whereas serine-163 is responsive to PKA activation.

## Discussion

Large-scale label-free quantitative phosphorylation analyses using phosphopeptide enrichment techniques have been demonstrated by various research groups; however, specific protein label-free quantitative phosphorylation analyses that combine immuno-precipitation SDS-PAGE separation, in-gel digestion and phosphopeptide enrichment techniques have not frequently been reported. Specifically, the reproducibility of the specific protein label-free quantitative phosphorylation analysis approach has not been systematically studied until now, which can be attributed to the somewhat laborious and time-consuming sample preparation procedure. Accordingly, most specific protein quantitative phosphorylation analysis studies have avoided using phosphopeptide enrichment techniques following the target protein immuno-precipitation or have employed isotope-labeling strategies to monitor the phosphorylation level changes under different conditions [22-25]. In this study, we demonstrated that the entire sample preparation procedure including immuno-precipitation, SDS-PAGE separation, in-gel digestion and IMAC phosphopeptide enrichment could be conducted in a reproducible manner for the subsequent label-free phosphopeptide quantification analysis. Normalization of the phosphopeptide peak areas using the internal standard bovine alpha casein phosphopeptide was expected to lower the level of peak area deviation that resulted from variable phosphopeptide enrichment efficiencies in the three independent experiments; however, a similar degree of peak area deviations was observed. The level of peak area deviation observed in this study (average relative standard deviation of 9.5%) is comparable to previously reported isotope labeling quantitative analysis results using SILAC, iTRAQ, and TMT techniques [26-28]. The relatively low peak area deviations in the repeated experiments suggested a high level of reproducibility, which is crucial for successful label-free quantitative phosphorylation analyses.

In this study, distinct phosphorylation changes of transgelin2 in Jurkat T cells under PKC and PKA activation conditions was monitored using our specific protein label-free quantitative phosphorylation analysis. The quantitative phosphorylation analysis of transgelin2 under these two conditions was expected to reveal the phosphorylation dynamics of transgelin2 during immune response activation and homeostasis. A total of six transgelin2 phosphorylation sites were identified in this study, and four phosphorylation sites exhibited specific kinase-dependent phosphorylation changes. Among the three phosphorylation sites showing PKC-dependent phosphorylation changes, threonine-180 and serine-185 are located in the calponin repeat domain, of which a physiological role has not yet been well described. However, Sugaya et al. reported that the calponin repeat domain 1 (CNR1: aa164-203) of smooth muscle calponin1 (CNN1) is more important with regard to the actin-binding ability than the previously reported actin-binding site (ABS: aa142-163) [5]. In their study, the phosphorylation of serine-175 located in CNR1 was mediated by PKC, and the phosphorylation status of this residue lowered the actin-binding of calponin1. Transgelin2 is very similar to calponin1 in terms of its tissue-specific expression pattern, actin-binding properties and structural domain compositions. Transgelin2 contains one calponin homology domain and one calponin repeat domain, whereas calponin1 contains two additional calponin repeat domains in the C-terminal. Although these two proteins share a sequence homology of 43%, all of the phosphorylation sites identified in this study except serine-163 are conserved and located in the same position in the sequence alignment analysis (Additional file 8: Figure S7). Serine-175 of CNN1, a PKC phosphorylation site, is aligned with serine-185 of transgelin2. PKC-dependent phosphorylation changes of threonine-180 have not been reported elsewhere. We hypothesize that the substantial phosphorylation changes of threonine 180th and serine 185th in CNR under PKC activation condition can greatly alter the actin-binding properties of transgelin2. Serine-11, which displayed PKC activation-dependent phosphorylation changes, does not belong to any well-defined structural domains. The potential functions of this phosphorylation site with regard to the actin-binding property of transgelin2 cannot be ignored; however, because the phosphorylation level was significantly increased. Further studies are required to investigate the functional significance of serine-11 phosphorylation on the actin binding of transgelin2. Notably, only one phosphorylation site, serine-163 showed PKA activation-dependent phosphorylation changes. Although the level of phosphorylation change for serine-163 was not as significant as expected, statistically significant differences (p < 0.05) were observed in three independent experiments. Serine-163 was positioned in the previously reported actin-binding site (ABS) of two close homologs of transgelin2, CNN1 and transgelin/SM22α.(CNN1: aa142-163, SM22α: aa151-166) The functional roles of this ABS with regard to actin-binding have been demonstrated by several domain deletion mutagenesis experiments with CNN1 and SM22α [2,5]. Additionally, the serine-163 phosphorylation-dependent actin-binding ability of transgelin2 was suggested by Leung et al [16]. Therefore, PKA-dependent serine-163 phosphorylation in the ABS may also modulate the actin binding of transgelin2. Taken together, multiple actin-binding regions of transgelin2 participate to accomplish its full actin-binding capability, and the actin-binding affinity of each actin-binding region appears to be modulated by specific kinase-dependent phosphorylation changes. Accordingly, different actin-binding properties or conflicting cellular functions of transgelin2 may result from distinct intracellular signaling events under immune response activation or homeostasis conditions.

## Conclusions

Our specific protein label-free phosphorylation quantitative analysis revealed distinct phosphorylation changes of transgelin2 in Jurkat T cell lines under the PKC and PKA activation conditions, which are comparable to immune cell activation and homeostasis conditions, respectively. Among the six transgelin2 phosphorylation sites identified in this study, four phosphorylation sites were located in three potential actin-binding regions: the ABS, the calponin homology domain (CH) and the CNR1. Notably, phosphorylation sites located in two actin-binding regions demonstrate specific kinase-dependent phosphorylation changes. Threonine-180 and serine-185, which are located in the CNR, exhibited significantly increased phosphorylation exclusively under the PKC activation conditions. Serine-163, which is located in the ABS, exhibited solely PKA-dependent phosphorylation changes. The direct involvement of these two regions during actin binding has been previously demonstrated by functional analyses of transgelin2 homologues. Because the CNR was more important than the ABS with regard to the actin binding, substantial phosphorylation changes of the CNR under PKC activation conditions could effectively attenuate any interactions between transgelin2 and cytoskeletal actin. These results suggest that different actin binding capabilities or two conflicting cellular functions of transgelin2 may be meticulously controlled by specific kinase cascades activated under immune response activation or immune response homeostasis conditions. Finally, our specific protein quantitative phosphorylation analysis method which can monitor the phosphorylation changes of individual phosphorylation sites under any specific kinase activation conditions is especially useful for functional validation of protein phosphorylation because more than one type of protein kinases is likely to be involved in regulating the functional state of a target protein.

## Methods

### Reagents

HPLC grade acetonitrile (ACN), methanol, water and BCA Protein assay kit were purchased from Fisher Scientific (Fair Lawn, NJ, USA). Fetal bovine serum (FBS), Geneticin (G418) and RPMI medium 1640 were purchased from GIBCO (Auckland, N.Z.). Pro-Q Diamond phosphoprotein gel staining kit was purchased from Molecular Probes (Eugene, OR, USA). Rabbit polyclonal anti-GFP was developed in rabbit by using purified recombinant full-length GFP protein (AbFrontier). All other chemicals were obtained from Sigma (St. Louis, MO, USA).

### Cell culture, Transfection and Phosphatase Inhibitor treatments

Jurkat T cells were transiently transfected with cDNAs encoding GFP-tagged transgelin2 by Nucleofector Kit V (Amaxa) according to manufacturer’s instructions and then selected with 1 mg/mL Geneticin. Jurkat T cells (TIB-152, American Type Culture Collection) transfected with GFP-tagged transgelin2 were grown in RPMI 1640 medium supplemented with 10% FBS and 1% penicillin/streptomycin at 37°C. Jurkat T cells expressing GFP-tagged transgelin2 were grown to confluence and then subjected to serum deprivation for 18 h. These cells were subjected to each of the following treatment conditions. No-activation condition samples were untreated or treated with 100 nM calyculin A (LC Laboratories), or 1 μM okadaic acid for 30 min prior to cell harvest. PKA activation condition was induced by 100 nM forskolin and PKC activation was induced by 200 nM phorbol 12-myristate 13-acetate (PMA) for 30 min. Two protein kinase activation condition samples were then treated with 100 nM calyculin A for 30 min prior to cell harvest.

### Cell lysis, Immuno-precipitation, SDS PAGE and In-gel digestion

No activation and kinase activation (PKA or PKC) cells were washed and harvested with cold phosphate buffered saline (PBS). The pellets were resuspended in lysis buffer (1% Nonidet P-40, 50 mM Tris–HCl pH 8.0, 150 mM NaCl and 2 mM EDTA) with protease inhibitor cocktail (Roche) and phosphatase inhibitor cocktail (Roche). The lysates were centrifuged at 13000 rpm for 10 min. The supernatant was transferred to a new tube. For immune-precipitation experiments, the immune complexes containing 5 mg proteins and anti-GFP conjugated Sepharose 4B (GE Healthcare) were gently mixed for overnight at 4°C and then washed twice with lysis buffer. Eluted proteins by heating of the beads at 95°C for 10 min in Laemmli SDS-PAGE sample buffer were separated by 10% SDS-PAGE. For phospho-specific fluorescent staining, all gels were treated with fixation solution (3 × 30 min), washed with deionized water (3 × 10 min), and stained with Pro-Q Diamond staining solution for 90 min. After Pro-Q Diamond staining, gels were imaged using a FLA-7000 imaging analyzer (Fujifilm). Following scanning of Pro-Q Diamond-stained gels, the gels were also stained with Coomassie Brilliant Blue (CBB) R250. The gel pieces were subjected to in-gel digestion as described previously [29]. For linearity experiments, various concentrations of bovine alpha casein samples ranging from 0.1 to 1.0 μg were added to the SDS-PAGE separation.

### IMAC phosphopeptide enrichment

A fused-silica capillary (250 μm i.d. × 360 μm o.d., Polymicro Technologies) was packed with 3 cm of 5 μm 120 Å ReproSil Pur Aqua C18 (Phenomenex) using a high-pressure Bomb for desalting purposes. The tryptic digest samples were loaded onto the column at a flow rate of 2 μL/min. The column was washed with 1 mL of 1% formic acid and 120 μL of IMAC binding buffer (40% ACN, 0.1% formic acid) was used to elute peptides. PHOS-Select iron affinity gel (15 μL of 50% beads slurry) was incubated with the desalted peptides for 1 hr at room temperature. After beads were washed with 1 mL of the IMAC binding buffer, bound phosphopeptides were eluted using 200 μL of IMAC elution buffer (200 mM NH_4_H_2_PO_4_) and the resulting phosphopeptide samples were analyzed by micro RPLC-MS/MS. Micro RPLC-MS/MS analyses were carried out only once for each phosphopeptide-enriched sample to show reproducibility of our label-free quantitative phosphorylation analysis.

### Micro RPLC-MS/MS analysis

Micro RPLC-MS/MS analysis was performed using an Agilent 1100 series high performance liquid chromatography (HPLC) pump (Agilent Technologies) coupled to a linear ion trap mass spectrometer (LTQ, Thermo Finnigan, San Jose, CA, USA) with an in-house manufactured nano-ESI interface. For micro RPLC-MS/MS analysis, samples were injected into a trap column (fused-silica capillary 250 μm i.d. × 360 μm o.d.; packed with 2 cm of Aqua C18) and separated with an analytical column (fused-silica capillary 100 μm i.d. × 360 μm o.d.; packed with 7.5 cm of Aqua C18). Buffer A (0.1% FA) and Buffer B (80% ACN, 0.1% formic acid) were used to elute bound peptides with a split flow system (flow rate: 250 nL/min) for 120 min linear gradient. In a positive ion mode, spectra were acquired with cycles of one full MS scan in the LTQ (m/z 400–2000) followed by 10 data-dependent MS/MS scans with normalized collision energy of 35% and dynamic exclusion time of 30 s.

### Data analysis

MS/MS spectra were searched against in-house database containing various transgelin2 homologues, GFP, IgG sequences and bovine alpha casein sequences using SEQUEST algorithm (Bioworks 3.2). Methionine oxidation and phosphorylation of serine, threonine, and tyrosine as variable modifications and carbamidomethylation of cysteine as a fixed modification were applied to the search. DTAselect (v.1.9) was used to filter the search results with the following criteria: fully tryptic digest ends, Xcorr > 1.8 for charge state 1+, Xcorr > 2.5 for charge state 2+ and Xcorr > 3.5 for charge state 3+. Assignments of the phosphopeptide sequences were further confirmed by manual validations on filtered MS/MS spectra. For the quantification of phosphorylation levels, selected ion chromatograms of identified phosphopeptides were constructed using Xcalibur 2.1.0 SP1 program (Thermo) and integrated peak areas were then calculated with a built-in feature of Xcalibur program for comparison purposes. Relative quantification of each phosphopeptide was performed by comparing peak-areas of no-activation condition and those of two kinase activation condition samples. Student’s *t* test was used for statistical analysis between no-activation condition and two kinase activation condition samples.

## Additional files

Additional file 1: Figure S1. Manually assigned MS/MS spectrum of a phosphopeptide containing threonine-84. Additional file 2: Table S1. Label-free quantitative phosphorylation analysis results of transgelin2 from three independent experiments. All peak areas were normalized to the co-added alpha casein tryptic digest peptide in the sample preparation. Additional file 3: Figure S2. Linearity of label-free quantitative phosphorylation analysis. Various concentrations of bovine alpha casein samples ranging from 0.1 to 1.0 μg were added in SDS-PAGE separation. Three independent experiments were performed and peak areas of individual alpha casein phosphopeptides were plotted against loaded amounts of alpha casein. Additional file 4: Figure S3. PKC-dependent phosphorylation changes of transgelin2 serine-11. Selected ion chromatograms of serine-11 containing phosphopeptide under no activation (A) and PKC activation (B) conditions. Manually assigned MS/MS spectrum of phosphopeptide containing serine-11 (C). Additional file 5: Figure S4. PKC dependent phosphorylation changes of transgelin2 threonine-180. Selected ion chromatograms of threonine-180 containing phosphopeptide under no activation (A) and PKC activation (B) conditions. Manually assigned MS/MS spectrum of phosphopeptide containing threonine-180 (C). Additional file 6: Figure S5. PKA-dependent phosphorylation changes of transgelin2 serine-163. Selected ion chromatograms of a phosphopeptide containing serine-163 under no activation (A) and PKA activation (B) conditions. Manually assigned MS/MS spectrum of phosphopeptide containing serine-163 (C). Additional file 7: Figure S6. Label-free relative quantitative phosphorylation analysis results of transgelin2 phosphorylation site (ser-185) under PKA and PKC activation conditions. * denotes the level of statistically significant difference in three independent sets of experiments. (p < 0.05). Additional file 8: Figure S7. Sequence alignment analysis results of transgelin2 and calponin1 using Protein BLAST feature from the PubMed website (www.ncbi.nlm.nih.gov/pubmed/).
