# Supplementary material for: Label-free quantitative phosphorylation analysis of human transgelin2 in Jurkat T cells reveals distinct phosphorylation patterns under PKA and PKC activation conditions
Source: Proteome Sci. 2015 Mar 26;13:14. doi: 10.1186/s12953-015-0070-9 (PMC4384351; doi:10.1186/s12953-015-0070-9)
Supplement: Additional file 1: Figure S1. — Manually assigned MS/MS spectrum of a phosphopeptide containing threonine-84. [file 12953_2015_70_MOESM1_ESM.pptx]

## Slide 1
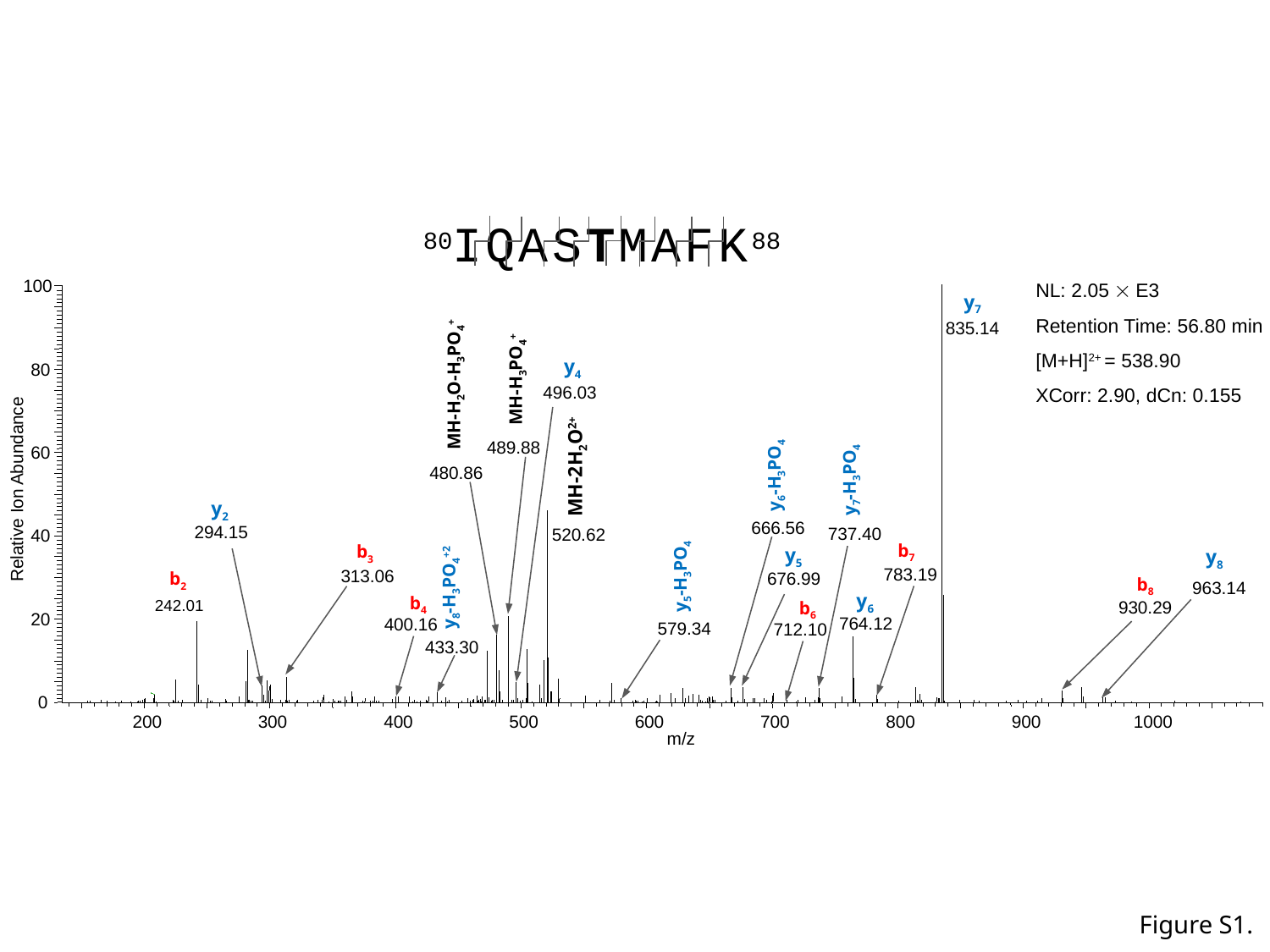

80IQASTMAFK88
NL: 2.05 × E3
Retention Time: 56.80 min
[M+H]2+ = 538.90
XCorr: 2.90, dCn: 0.155
100
y7
835.14
y4
80
MH-H3PO4+
MH-H2O-H3PO4+
496.03
489.88
60
MH-2H2O2+
y6-H3PO4
y7-H3PO4
480.86
Relative Ion Abundance
y2
666.56
294.15
737.40
520.62
40
b7
b3
y5
y8
y5-H3PO4
b2
783.19
313.06
b8
676.99
y8-H3PO4+2
963.14
y6
b4
b6
242.01
930.29
20
764.12
400.16
579.34
712.10
433.30
0
200
300
400
500
600
700
800
900
1000
m/z
Figure S1.
