# Supplementary material for: Label-free quantitative phosphorylation analysis of human transgelin2 in Jurkat T cells reveals distinct phosphorylation patterns under PKA and PKC activation conditions
Source: Proteome Sci. 2015 Mar 26;13:14. doi: 10.1186/s12953-015-0070-9 (PMC4384351; doi:10.1186/s12953-015-0070-9)
Supplement: Additional file 2: Table S1. — Label-free quantitative phosphorylation analysis results of transgelin2 from three independent experiments. All peak areas were normalized to the co-added alpha casein tryptic digest peptide in the sample preparation. [file 12953_2015_70_MOESM2_ESM.pptx]

## Slide 1
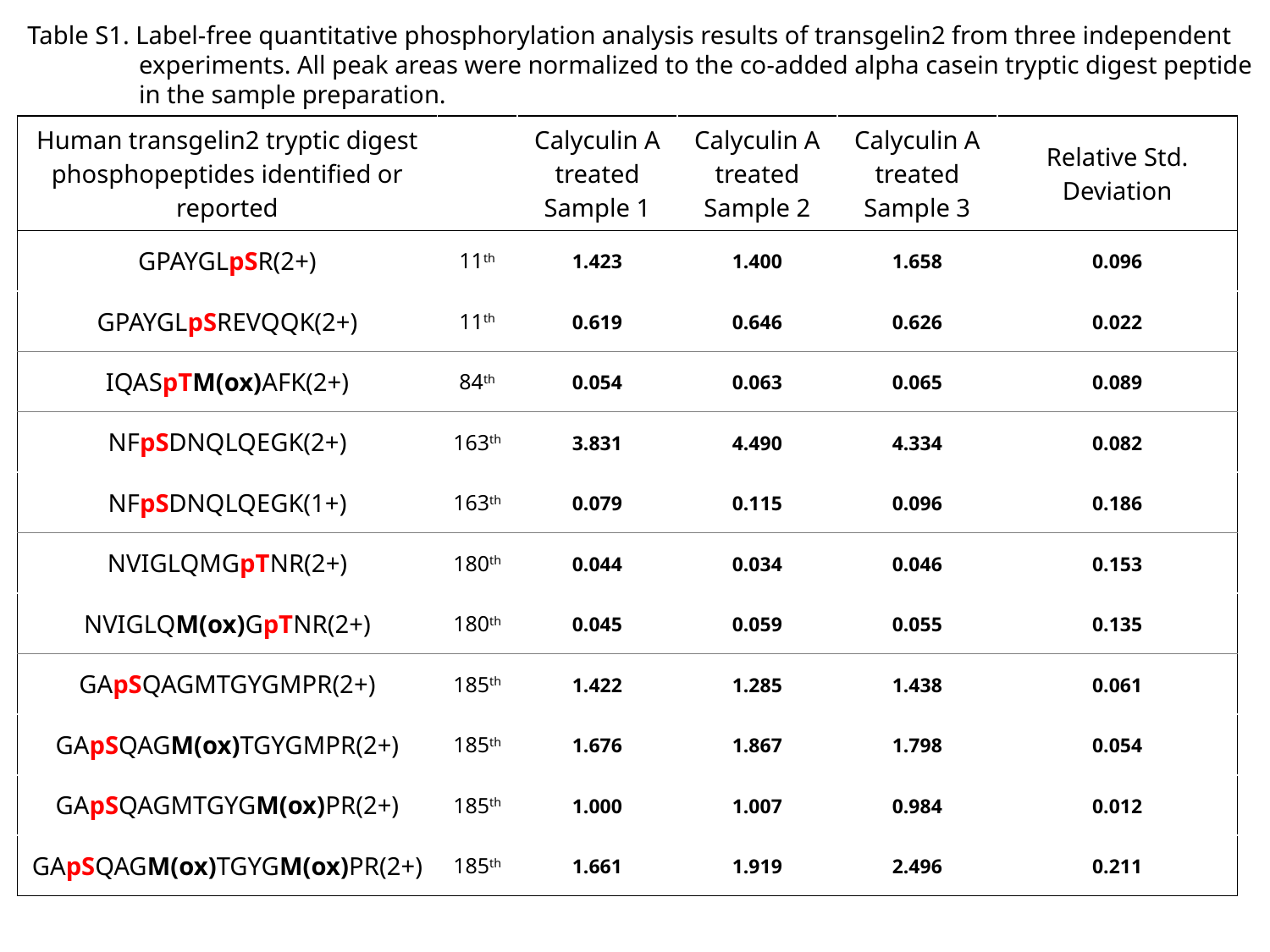

Table S1. Label-free quantitative phosphorylation analysis results of transgelin2 from three independent experiments. All peak areas were normalized to the co-added alpha casein tryptic digest peptide in the sample preparation.
| Human transgelin2 tryptic digest phosphopeptides identified or reported | | Calyculin A treated Sample 1 | Calyculin A treated Sample 2 | Calyculin A treated Sample 3 | Relative Std. Deviation |
| --- | --- | --- | --- | --- | --- |
| GPAYGLpSR(2+) | 11th | 1.423 | 1.400 | 1.658 | 0.096 |
| GPAYGLpSREVQQK(2+) | 11th | 0.619 | 0.646 | 0.626 | 0.022 |
| IQASpTM(ox)AFK(2+) | 84th | 0.054 | 0.063 | 0.065 | 0.089 |
| NFpSDNQLQEGK(2+) | 163th | 3.831 | 4.490 | 4.334 | 0.082 |
| NFpSDNQLQEGK(1+) | 163th | 0.079 | 0.115 | 0.096 | 0.186 |
| NVIGLQMGpTNR(2+) | 180th | 0.044 | 0.034 | 0.046 | 0.153 |
| NVIGLQM(ox)GpTNR(2+) | 180th | 0.045 | 0.059 | 0.055 | 0.135 |
| GApSQAGMTGYGMPR(2+) | 185th | 1.422 | 1.285 | 1.438 | 0.061 |
| GApSQAGM(ox)TGYGMPR(2+) | 185th | 1.676 | 1.867 | 1.798 | 0.054 |
| GApSQAGMTGYGM(ox)PR(2+) | 185th | 1.000 | 1.007 | 0.984 | 0.012 |
| GApSQAGM(ox)TGYGM(ox)PR(2+) | 185th | 1.661 | 1.919 | 2.496 | 0.211 |
