# Supplementary material for: Label-free quantitative phosphorylation analysis of human transgelin2 in Jurkat T cells reveals distinct phosphorylation patterns under PKA and PKC activation conditions
Source: Proteome Sci. 2015 Mar 26;13:14. doi: 10.1186/s12953-015-0070-9 (PMC4384351; doi:10.1186/s12953-015-0070-9)
Supplement: Additional file 3: Figure S2. — Linearity of label-free quantitative phosphorylation analysis. Various concentrations of bovine alpha casein samples ranging from 0.1 to 1.0 μg were added in SDS-PAGE separation. Three independent experiments were performed and peak areas of individual alpha casein phosphopeptides were plotted against loaded amounts of alpha casein. [file 12953_2015_70_MOESM3_ESM.pptx]

## Slide 1
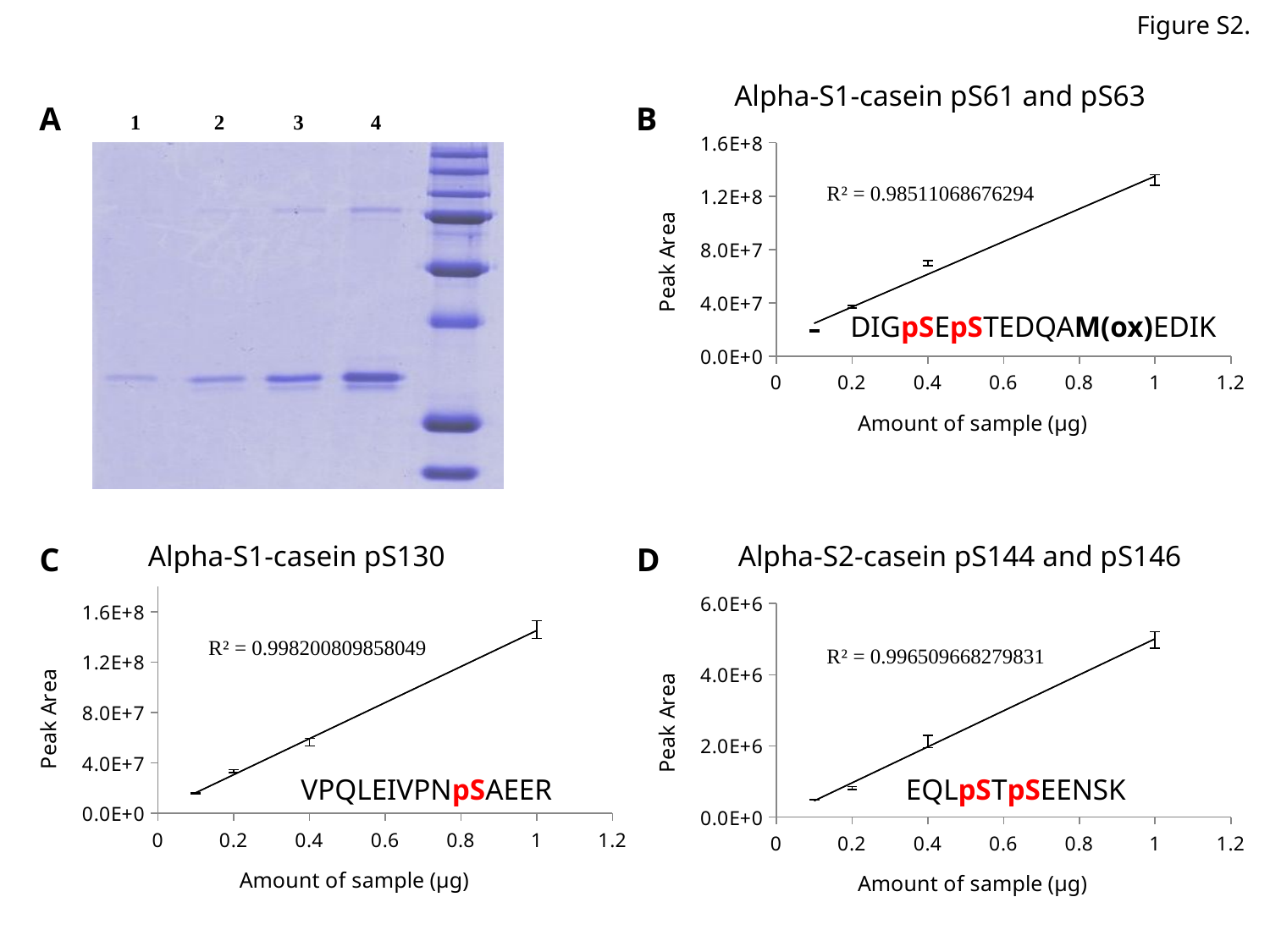

Figure S2.
Alpha-S1-casein pS61 and pS63
A
B
4
3
1
2
### Chart
| Category | Peak area |
|---|---|
DIGpSEpSTEDQAM(ox)EDIK
Alpha-S1-casein pS130
Alpha-S2-casein pS144 and pS146
C
D
### Chart
| Category | Peak area |
|---|---|
### Chart
| Category | Peak area |
|---|---|VPQLEIVPNpSAEER
EQLpSTpSEENSK
