# Supplementary material for: Label-free quantitative phosphorylation analysis of human transgelin2 in Jurkat T cells reveals distinct phosphorylation patterns under PKA and PKC activation conditions
Source: Proteome Sci. 2015 Mar 26;13:14. doi: 10.1186/s12953-015-0070-9 (PMC4384351; doi:10.1186/s12953-015-0070-9)
Supplement: Additional file 4: Figure S3. — PKC-dependent phosphorylation changes of transgelin2 serine-11. Selected ion chromatograms of serine-11 containing phosphopeptide under no activation (A) and PKC activation (B) conditions. Manually assigned MS/MS spectrum of phosphopeptide containing serine-11 (C). [file 12953_2015_70_MOESM4_ESM.pptx]

## Slide 1
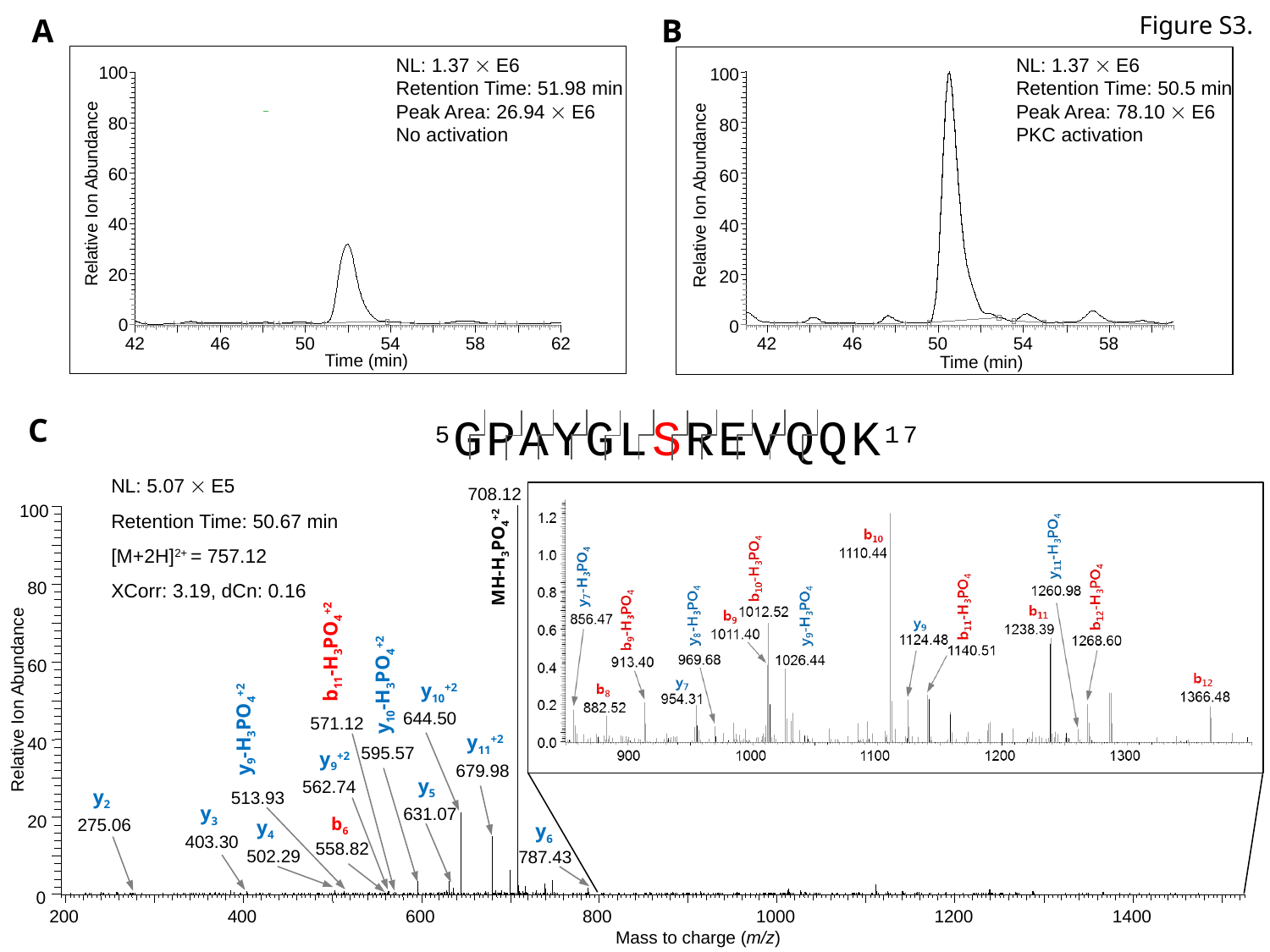

Figure S3.
A
B
NL: 1.37 × E6
Retention Time: 51.98 min
Peak Area: 26.94 × E6
No activation
NL: 1.37 × E6
Retention Time: 50.5 min
Peak Area: 78.10 × E6
PKC activation
100
100
80
80
60
60
Relative Ion Abundance
Relative Ion Abundance
40
40
20
20
0
0
42
46
50
54
58
62
42
46
50
54
58
Time (min)
Time (min)
5GPAYGLSREVQQK17
C
NL: 5.07 × E5
Retention Time: 50.67 min
[M+2H]2+ = 757.12
XCorr: 3.19, dCn: 0.16
708.12
100
MH-H3PO4+2
80
b11-H3PO4+2
60
y10-H3PO4+2
y10+2
Relative Ion Abundance
644.50
y9-H3PO4+2
571.12
y11+2
40
y9+2
595.57
679.98
y5
562.74
y2
513.93
y3
631.07
b6
y4
20
y6
275.06
403.30
558.82
502.29
787.43
0
200
400
600
800
1000
1200
1400
Mass to charge (m/z)
