# Supplementary material for: Label-free quantitative phosphorylation analysis of human transgelin2 in Jurkat T cells reveals distinct phosphorylation patterns under PKA and PKC activation conditions
Source: Proteome Sci. 2015 Mar 26;13:14. doi: 10.1186/s12953-015-0070-9 (PMC4384351; doi:10.1186/s12953-015-0070-9)
Supplement: Additional file 5: Figure S4. — PKC dependent phosphorylation changes of transgelin2 threonine-180. Selected ion chromatograms of threonine-180 containing phosphopeptide under no activation (A) and PKC activation (B) conditions. Manually assigned MS/MS spectrum of phosphopeptide containing threonine-180 (C). [file 12953_2015_70_MOESM5_ESM.pptx]

## Slide 1
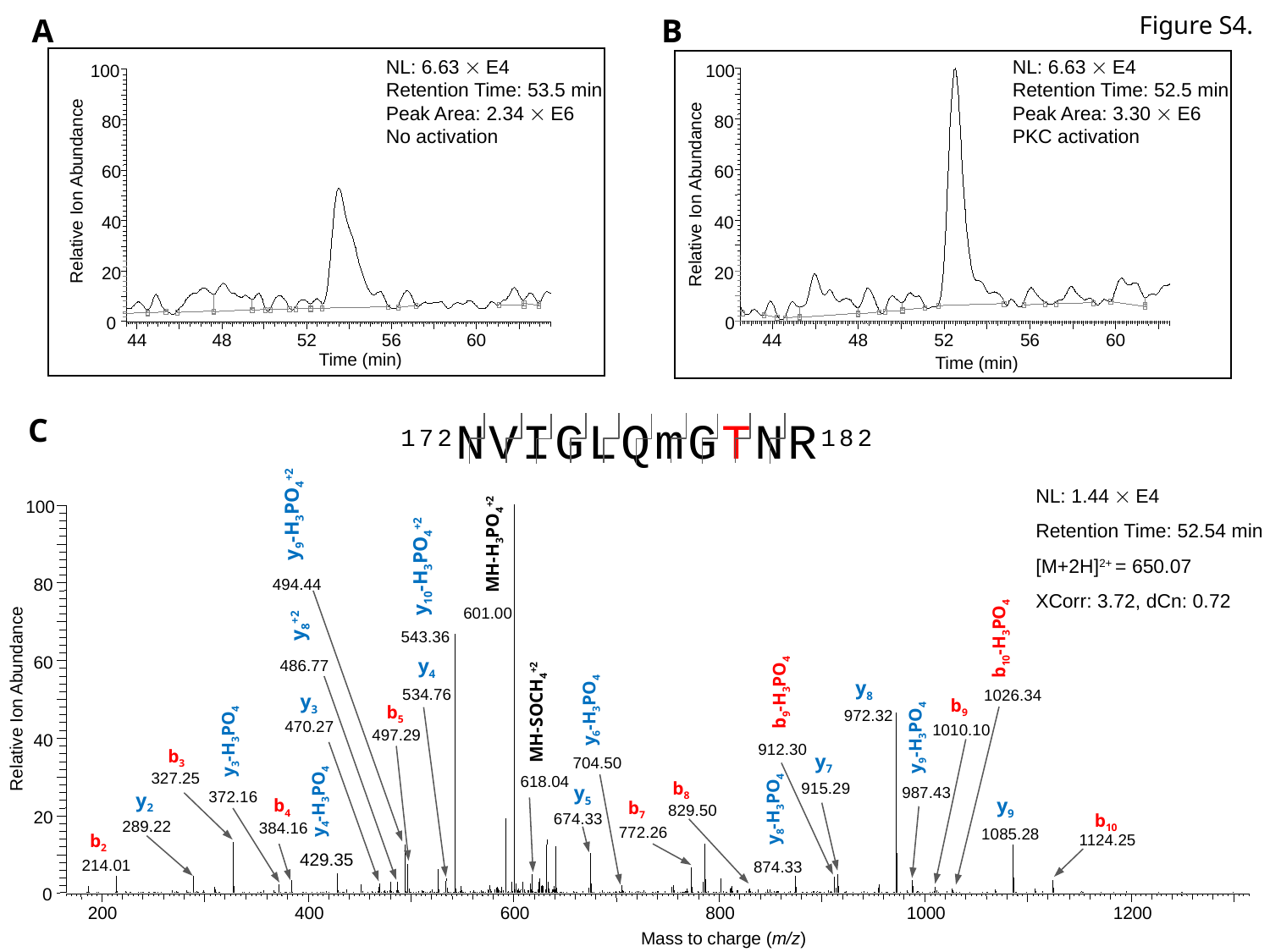

Figure S4.
A
B
NL: 6.63 × E4
Retention Time: 53.5 min
Peak Area: 2.34 × E6
No activation
NL: 6.63 × E4
Retention Time: 52.5 min
Peak Area: 3.30 × E6
PKC activation
100
100
80
80
60
60
Relative Ion Abundance
Relative Ion Abundance
40
40
20
20
0
0
44
48
52
56
60
44
48
52
56
60
Time (min)
Time (min)
172NVIGLQmGTNR182
C
NL: 1.44 × E4
Retention Time: 52.54 min
[M+2H]2+ = 650.07
XCorr: 3.72, dCn: 0.72
y9-H3PO4+2
100
MH-H3PO4+2
y10-H3PO4+2
80
494.44
601.00
y8+2
b10-H3PO4
543.36
y4
60
486.77
y8
b9-H3PO4
y3
534.76
1026.34
b9
Relative Ion Abundance
y6-H3PO4
b5
MH-SOCH4+2
972.32
470.27
y9-H3PO4
1010.10
y3-H3PO4
497.29
40
b3
912.30
y7
704.50
327.25
b8
618.04
y5
915.29
y2
y4-H3PO4
987.43
372.16
y9
b4
y8-H3PO4
b7
829.50
b10
20
674.33
289.22
384.16
772.26
b2
1085.28
1124.25
429.35
214.01
874.33
0
200
400
600
800
1000
1200
Mass to charge (m/z)
