# Supplementary material for: Label-free quantitative phosphorylation analysis of human transgelin2 in Jurkat T cells reveals distinct phosphorylation patterns under PKA and PKC activation conditions
Source: Proteome Sci. 2015 Mar 26;13:14. doi: 10.1186/s12953-015-0070-9 (PMC4384351; doi:10.1186/s12953-015-0070-9)
Supplement: Additional file 6: Figure S5. — PKA-dependent phosphorylation changes of transgelin2 serine-163. Selected ion chromatograms of a phosphopeptide containing serine-163 under no activation (A) and PKA activation (B) conditions. Manually assigned MS/MS spectrum of phosphopeptide containing serine-163 (C). [file 12953_2015_70_MOESM6_ESM.pptx]

## Slide 1
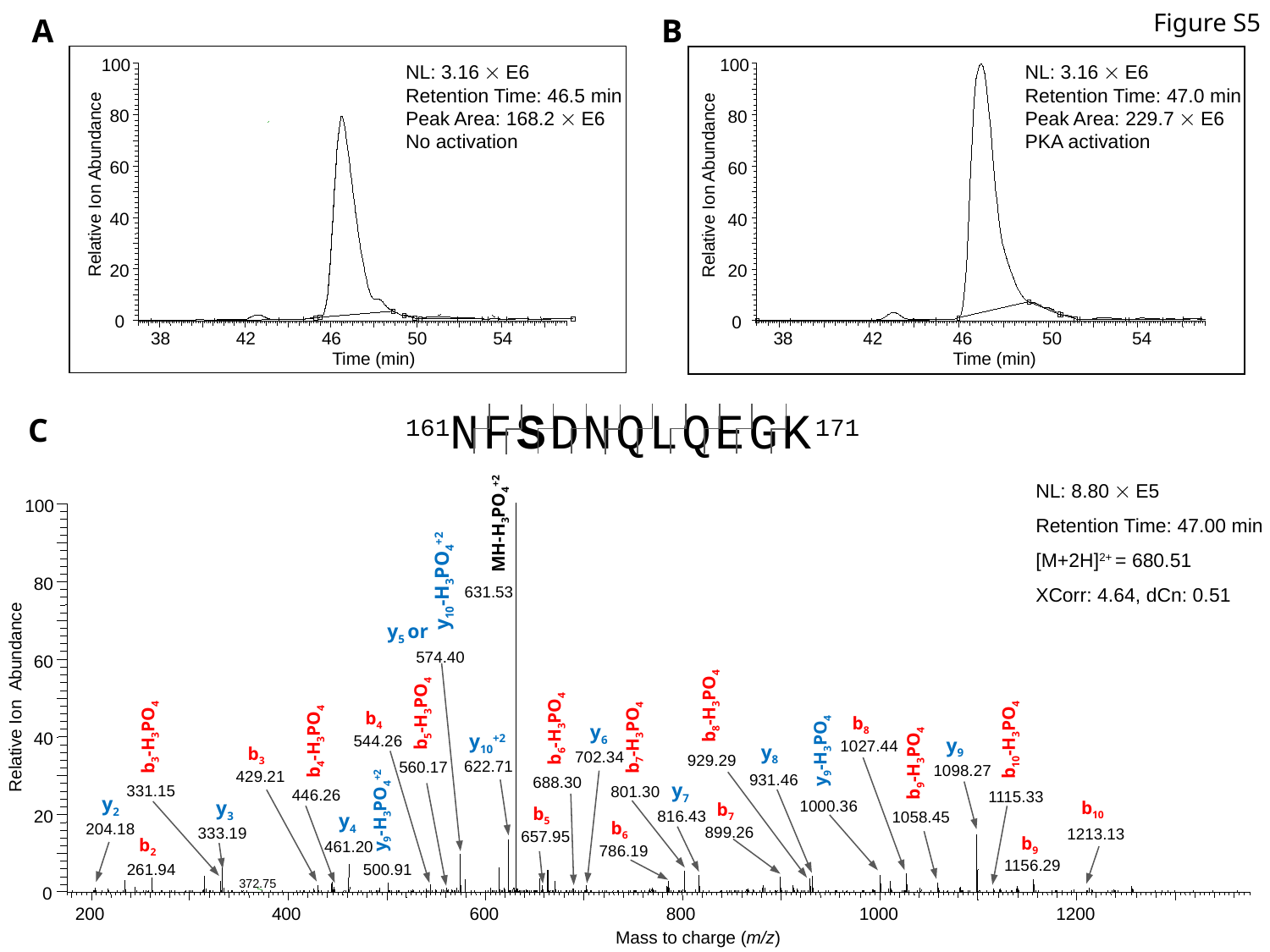

Figure S5
A
B
100
100
NL: 3.16 × E6
Retention Time: 46.5 min
Peak Area: 168.2 × E6
No activation
NL: 3.16 × E6
Retention Time: 47.0 min
Peak Area: 229.7 × E6
PKA activation
80
80
60
60
Relative Ion Abundance
Relative Ion Abundance
40
40
20
20
0
0
38
42
46
50
54
38
42
46
50
54
Time (min)
Time (min)
161NFSDNQLQEGK171
C
NL: 8.80 × E5
Retention Time: 47.00 min
[M+2H]2+ = 680.51
XCorr: 4.64, dCn: 0.51
100
MH-H3PO4+2
y10-H3PO4+2
y5 or
80
631.53
574.40
60
Relative Ion Abundance
b8-H3PO4
b5-H3PO4
b4
b8
b6-H3PO4
y6
b3-H3PO4
b7-H3PO4
b10-H3PO4
b4-H3PO4
y10+2
y9
40
544.26
y9-H3PO4
y8
1027.44
b3
b9-H3PO4
702.34
929.29
622.71
560.17
1098.27
429.21
931.46
y7
688.30
331.15
801.30
446.26
y2
1115.33
y3
b10
b7
y9-H3PO4+2
1000.36
b5
y4
20
816.43
1058.45
b6
204.18
899.26
333.19
1213.13
b9
657.95
b2
461.20
786.19
1156.29
261.94
500.91
372.75
0
200
400
600
800
1000
1200
Mass to charge (m/z)
