# Supplementary material for: Label-free quantitative phosphorylation analysis of human transgelin2 in Jurkat T cells reveals distinct phosphorylation patterns under PKA and PKC activation conditions
Source: Proteome Sci. 2015 Mar 26;13:14. doi: 10.1186/s12953-015-0070-9 (PMC4384351; doi:10.1186/s12953-015-0070-9)
Supplement: Additional file 7: Figure S6. — Label-free relative quantitative phosphorylation analysis results of transgelin2 phosphorylation site (ser-185) under PKA and PKC activation conditions. * denotes the level of statistically significant difference in three independent sets of experiments. (p < 0.05). [file 12953_2015_70_MOESM7_ESM.pptx]

## Slide 1
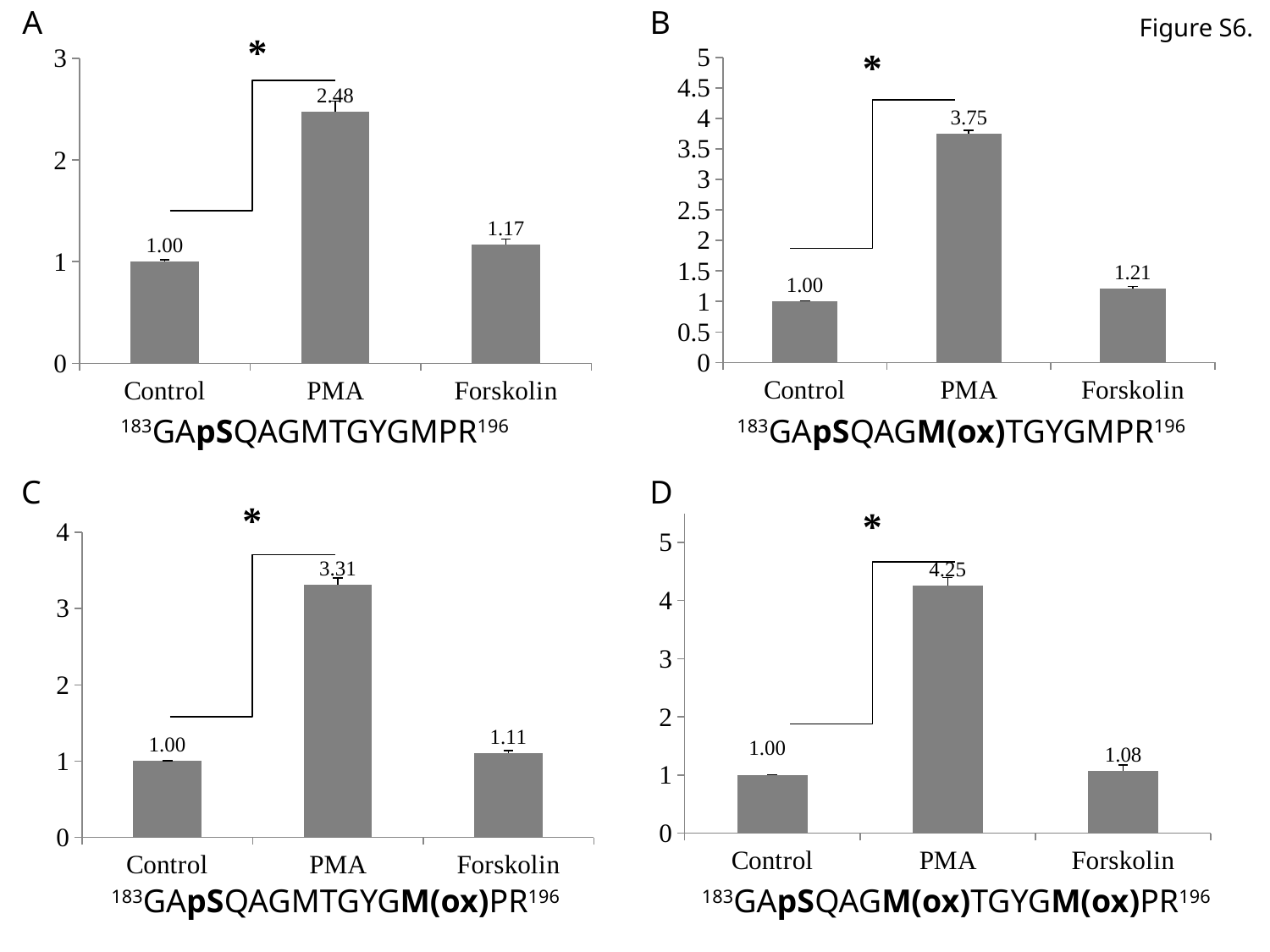

A
B
Figure S6.
*
### Chart
| Category | Cal |
|---|---|
| Control | 1.0 |
| PMA | 3.7547447148847866 |
| Forskolin | 1.214992866715663 |
### Chart
| Category | Cal |
|---|---|
| Control | 1.0 |
| PMA | 2.4764794299393196 |
| Forskolin | 1.169849134331682 |*
183GApSQAGMTGYGMPR196
183GApSQAGM(ox)TGYGMPR196
C
D
*
*
### Chart
| Category | Cal |
|---|---|
| Control | 1.0 |
| PMA | 4.252139515423529 |
| Forskolin | 1.0767943402685332 |
### Chart
| Category | Cal |
|---|---|
| Control | 1.0 |
| PMA | 3.3106960950764 |
| Forskolin | 1.1061120543293719 |183GApSQAGMTGYGM(ox)PR196
183GApSQAGM(ox)TGYGM(ox)PR196
