# Supplementary material for: Label-free quantitative phosphorylation analysis of human transgelin2 in Jurkat T cells reveals distinct phosphorylation patterns under PKA and PKC activation conditions
Source: Proteome Sci. 2015 Mar 26;13:14. doi: 10.1186/s12953-015-0070-9 (PMC4384351; doi:10.1186/s12953-015-0070-9)
Supplement: Additional file 8: Figure S7. — Sequence alignment analysis results of transgelin2 and calponin1 using Protein BLAST feature from the PubMed website (www.ncbi.nlm.nih.gov/pubmed/). [file 12953_2015_70_MOESM8_ESM.pptx]

## Slide 1
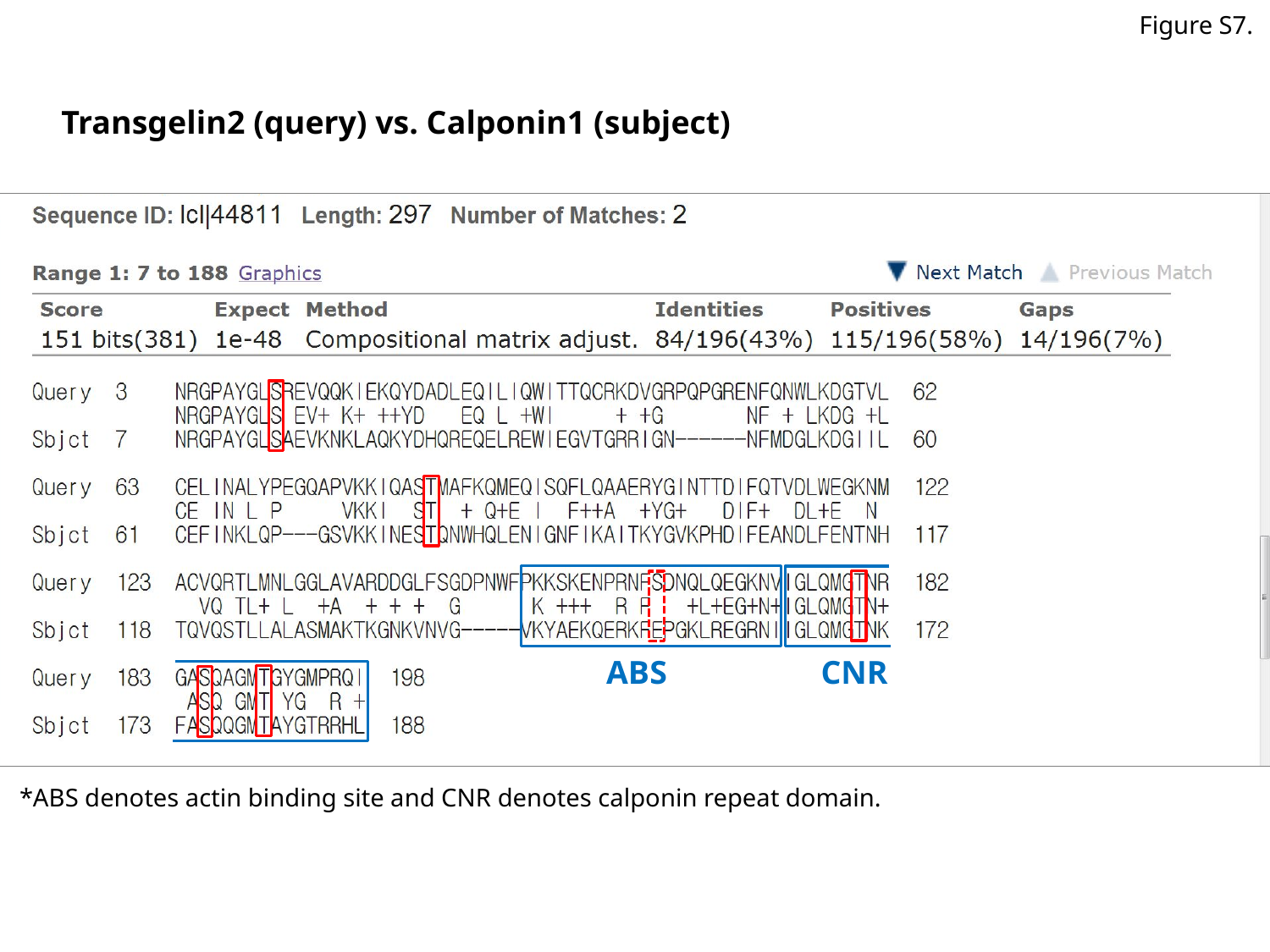

Figure S7.
Transgelin2 (query) vs. Calponin1 (subject)
ABS
CNR
*ABS denotes actin binding site and CNR denotes calponin repeat domain.
